# The market for reproductive tourism: an analysis with special reference to Greece

**DOI:** 10.1186/s41256-017-0037-8

**Published:** 2017-06-12

**Authors:** Anastasia Paraskou, Babu P. George

**Affiliations:** 1 CROSSBEES Healthcare Consultancy, Greece, 48 Themistokli Dervi Av, CY-1066 Nicosia, Cyprus; 2 Fort Hays State University, Hays, KS 67601 USA; 3 Swiss Management Center University, Vorstadt 26a, 6300 Zug, Switzerland

**Keywords:** Medical tourism, Reproductive tourism, Assisted reproduction, Competition, Strategy, Greece

## Abstract

**Background:**

For many people, the need for parenthood remains unfulfilled due to biological reasons and a remedy for these individuals is assisted reproduction (AR). Because of widely differing and sometimes incompatible legislations around the world related to AR, there is considerable confusion across national borders. Within Europe, Greece seems to be in a comparatively favorable position because of lower restrictions and the availability of decent quality specialized medical facilities. This research is a market study with a business perspective and explores the emerging landscape of reproductive tourism (RT) in Greece.

**Methods:**

The research adopted mixed methods. First, open-ended questions were used to interview foreign medical tourists and staff in various AR clinics. Based on the insights from these interviews and guided by the extent literature, a survey instrument was prepared and administered among 130 patients.

**Results:**

Findings indicate that Greece still lack policies that nurture transparency and dynamic response to technological changes in AR. Also, the travel industry lack specialists who can effectively liaison with clinics, who understand the availability of AR technologies, regulations, and the unique needs of AR tourists.

**Conclusions:**

Globally, the need for assisted reproduction has tremendously increased; yet, the supply of facilities is lagging far behind. There is a unique advantage for clinics located in touristic locations in countries that offer cheaper treatment options. Given the shape of its debt-ridden economy, Greece needs foreign exchange inflows and gaining first mover advantage in reproductive tourism is probably an important way to achieve this. This research draws up a reproductive tourism strategy for Greece.

## Background

Assisted reproduction had become considerably more popular over the last three decades, due to scientific progress [3, 18]. Changing preferences as to an individual’s lifestyle, societal pressures, and greatly enhanced availability of information are counting prominently among them [6, 20, 31]. People seeking assisted reproduction (AR) have become older, more numerous, and better informed [21, 28]. Cross-border travel for assisted reproduction is a natural progression of this trend.

The ability of more people to travel more far for less is contributing to what is the broader framework of this research: medical tourism [7, 16]. This term comprises every form of travelling – mainly, albeit not necessarily, going abroad – for the purpose of medical treatment [14, 15]. This includes standard medicinal procedures, but also plastic surgery, a stay in a health resort, or assisted reproduction [26]. The second component of medical tourism – tourism – indicates a desire to not just visit a facility or to hospitalize oneself, but to undergo treatment in circumstances which by themselves contribute to one’s well-being [5, 27]. If the properties of the destination were meaningless for the patient, the more adequate term would be medical travel [29].

Reproductive tourism within the context of medical tourism has many important and significant aspects that should be discussed and elaborated [9, 12, 13, 31]. Given that the key reasons for these travels are legislative restrictions in their own country, accessibility, treatment costs and an improved quality of treatment [35–38], the European Society of Human Reproduction and Embryology came to the conclusion that “tourism” is not the primary concern of these patients, but rather “cross-border care.” The term “cross-border reproductive care” (CBRC) has therefore been adopted to describe people who cross borders to receive fertility treatment.

**Fig. 1:**
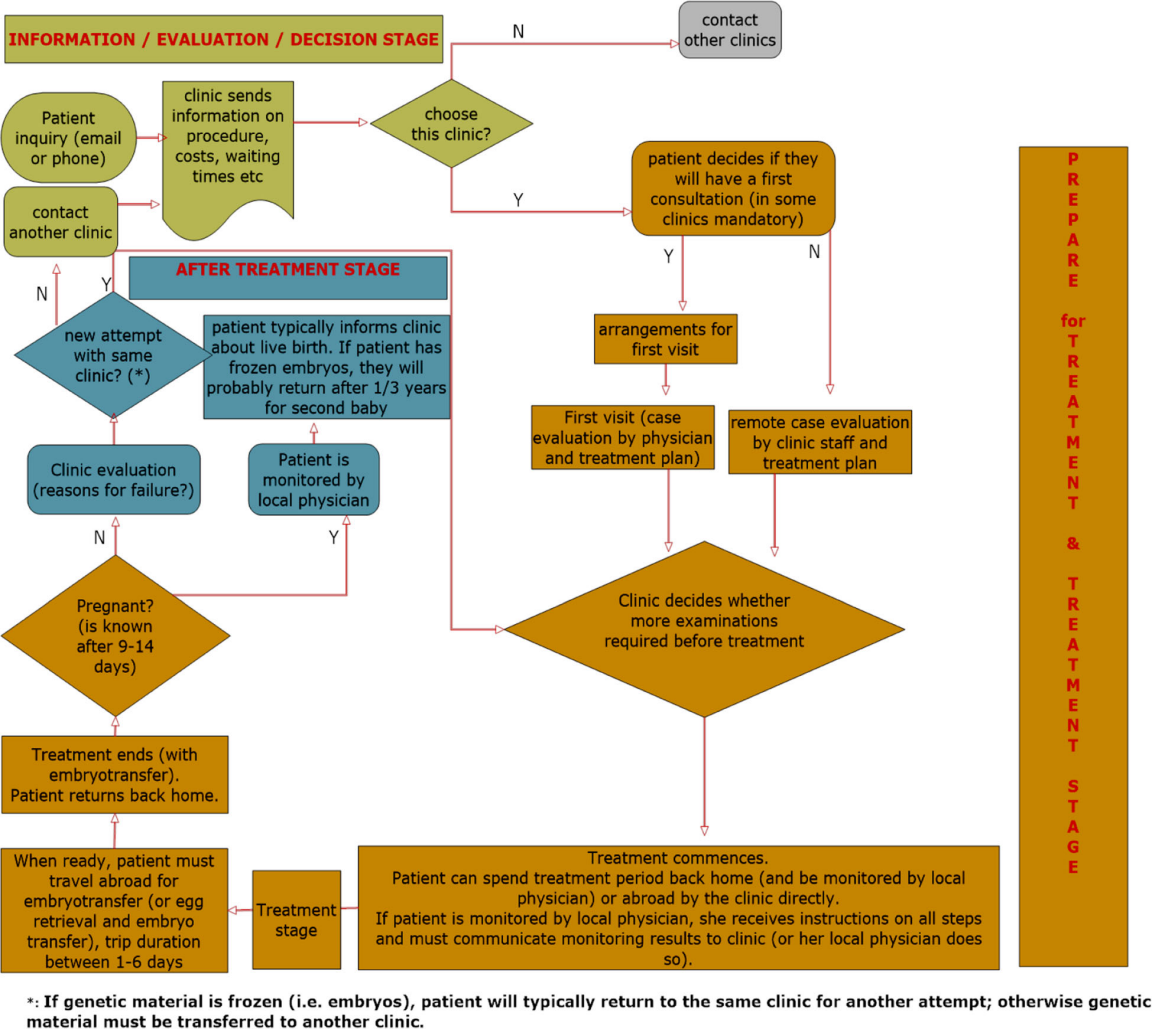
Stages involved in the treatment of ART abroad. Assisted Reproduction Decision Process Flow

The flow of reproductive tourism, from the perspective of the patient-tourists, is as seen in Fig. 1.

Table 1 is an approximated summary of the cost of assisted reproduction treatment across selected countries. Note that the cost associated with tourism for the patient or those accompanying is not included in these figures. The amounts refer to costs for self-payers and do not cover additional examinations, lab techniques, or fees for cryopreservation of genetic material. Average costs derived from inquiries to 3-5 clinics in each country between 2011 and 2013.

**Table 1:** An international comparison of the cost of assisted reproduction

Country	Indicatory average treatment costs in Europe & the USA (in EUR)			
	IVF (1)	Oocyte donation (2)	Embryo donation (3)	Preimplantation Genetic Screening (4)
UK	5800	14000	7000	4000
Germany	3000	not allowed	not allowed	(partially allowed): 3500
Italy	6000	not allowed (5)	not allowed (5)	(partially allowed): 3500
Denmark (6)	2500	5000	not allowed	3500
Spain	5000	9000	6000	4500
Czech Republic	1900	5000	2000	3000
Russia	2000	8000	5000	4000
Ukraine	1800	6000	3000	2000
Greece	3500	6000	3000	3000
Cyprus	3000	6000	3000	3000
USA	2000	15000	8000	6000

*Notes*: Rates in local currency, converted in EUR on xe.com in August 2013.

(1) Intended is IVF with intracytoplasmic sperm injection (ICSI).

(2) Costs refer to oocyte donation using an exclusive female donor; in some countries, e.g. UK, there are shared programs (i.e., one female donor donates to more recipients, or a woman undergoing treatment herself, donates a part of her oocytes to another woman). Such programs cost less.

(3) There is a difference between embryo donation (which implies using embryos resulting from a female and a male donor) and embryo adoption (which implies the use of embryos left over from couples who do not need them any longer). Legislation in this regard varies among the countries. The costs in this table concern embryo donation.

(4) There are two methods for aneuploidy screening: The FISH method (fluorescence in situ hybridization) and CGH array (comparative genomic hybridization). This table refers to CGH array and the fees are based on an assumption of eight embryos.

(5) Legislation has meanwhile changed.

(6) Denmark does not allow donation of an embryo, only oocytes and sperm can be donated but one of the intended parents always have to be genetically related to the child.

Source(s): Compiled based on data from World Tourism Organization, *ESHRE* European Society of Human Reproduction and Embryology, *HFEA* Human Fertilization and Embryology Authority; & Connolly et al. [12]

It was interesting to observe that many countries have policies of partial or full cost reimbursement. Table 2 given below highlights some international diversity in this regard:

**Table 2:** Reimbursement Policies in Selected Countries

Country	Insurance Coverage
Austria	Approximately two thirds of costs covered.
Australia	Medicare covers almost all costs, the difference “out-of-pocket” costs. Must be paid by patient. Additional funds possible through EMSN (Extended Medicare Safety Net).
Belgium	Patient pays 5–10%.
	In initial treatments, only SET covered.
Czech Republic	Up to 4 cycles, age limit for female is 47 years.
Denmark	Up to 3 cycles. Free fertility treatment only at a public clinic, to have the first child of a couple. If you want more children you have to pay the full price for treatment in a private practice. Also, the woman cannot be more than 40 years.
France	Some limitations in egg and sperm donation apply, otherwise complete coverage of 4 attempts.
Germany	50% coverage. Reimbursement granted only to married couples and up to the age of 40 years (female age). Private insurance regulations vary.
Italy	Coverage only for treatment in public centers.
	Differences between regions apply.
Israel	Full coverage until the birth of two children.
Korea	up to 3 cycles to married couples below 44 years and depending on the family income.
Spain	Reimbursement only for treatment in public centers. Egg and sperm donation not covered.
UK	Several criteria must be met for NHS funding of up to 3 cycles, such as: Female age may be up to 39 years; female must have a regular BMI. Further criteria are years and reasons for infertility, number of previous cycles and whether the patient has other children. The “postcode lottery” means that a woman may be eligible for treatment in any clinic of the country. If she refuses treatment, she is dropped out for lifetime.
USA	Couples must be ready to undergo treatment as soon as 10 weeks upon approval. Regulations vary. 14 states provide partial coverage and some 5 states provide full coverage.

Source: Published data on the websites of Public Health Departments of respective countries

This study examines circumstances which create incentives for reproductive tourism in the special context of Greece. The demand and the supply side of assisted reproduction as a business area will be addressed to provide a better understanding of this still emerging phenomenon. The legal framework that govern the transactions will also be discussed. An overview of the market conditions shows that Greece is endowed with considerable advantages. Our empirical investigation is expected to throw more light upon the existing understanding of reproductive tourism. Greece’s thoroughly liberal AR legislation, its advantageous position as an EU member state, endowed with natural beauties and perceived as part of the Christian Occident, may turn this country into a favorable location for foreigners seeking help for fertility problems.

The specific research questions that we choose to address are:

- What are some of the key opportunities and challenges for assisted reproductive tourism, in general and for Greece in particular?
- What are the perceptions of customers and service providers about the contemporary practice of reproductive tourism in Greece?
- What is the policy framework required to strengthen the competitiveness of reproductive tourism in Greece?

## Methods

This research was conducted in the tradition of mixed method of research. Since exploration of a largely uncharted field, i.e. reproductive tourism, was our primary objective, a preliminary stage involving qualitative inquiry was found to be appropriate. However, in addition to open ended qualitative interviews, we also used a questionnaire based survey to elicit quantifiable data related to some of the issues surrounding assisted reproduction and reproductive tourism. The questionnaire consisted of 30 questions that aimed at covering the following topics:

- Demographics of AR tourists
- Motivations for AR
- Relative positioning of Greece Vs other AR destinations
- Factors affecting the decision to choose Greece
- Perceived service quality in clinical and nonclinical settings

The sample used in the field research consisted of two groups of individuals: reproductive tourists and those representing the reproductive tourism supply side. Even though the geographical focus on this research was Northern Greece in the region around Thessaloniki, it was not strictly limited to this area. Persons interviewed were foreigners visiting other regions of Greece, too, for the purpose of obtaining assisted reproduction treatment. The physicians, embryologists, and other staff members at reproductive clinics in Greece who we interviewed practiced at different parts of the country.

The first group who we interviewed included with managers, owners, and assisted reproduction clinic personnel (general physicians, gynecologists, embryologists, geneticists, nurses, and administrative staff), identified on convenience sampling basis. Addresses were sourced from a public directory. Fourteen participants were interviewed with open ended questions. These individuals were asked to share their opinions about reproductive tourism in Greece, patient expectations in general, and the perceived quality which Greek medical clinics and hospitals offer to medical tourists. The industry experts provided answers in relation to the structure and functioning of specific medical and reproductive tourism features in Greece, as well as the opportunities and obstacles it is confronted with.

The second group consisted of foreigners coming to Greece seeking reproduction related treatments. Responses were collected directly from the patients who have had their treatment through an agency which was acting as a coordinator for foreigners seeking reproductive treatments in Greece. From that list provided by the agency, using a random number generator, we identified our respondents. Confidentiality was assured in writing. The questionnaire was presented to more than 300 people and the number of usable responses received was 130. Considering that for 2013 approximately 1000 individuals visited Greece as patients/customers for reproductive care [33], the sample size is around 13% of the population of reproductive medical tourists.

A confirmatory factor analysis that we performed upon the data yielded factors determining consumer choice. Based on the open ended qualitative comments, content analysis of the literature, and various secondary data sources, a SWOT analysis was performed. The policy framework needed for the growth of Greece in Reproductive Tourism (RT) was derived by triangulating the findings with the literature.

## Results

This section presents the findings of the study, based on the data gathered from the medical professionals and the patients. These results are independently presented and then cross-analyzed, in the light of the extant literature.

### Analysis of data gathered from the medical professionals

From our open-ended interviews with the industry’s supply side representatives, we gained feedback indicating that the medical staff were showing a favorable attitude towards their immediate working environment and to the economic prospects of Greece in RT. Physicians regarded themselves as honest and committed to the patients’ desire to achieve parenthood. As to the community of physicians working in AR, the interviewees frequently lashed out against colleagues who, in their opinion, mainly went after money, were partially or fully incompetent, and lacked honesty in their dealings with patients.

This negative attitude was in line with great discomfort vis-à-vis the institutional and legal circumstances of assisted reproduction in Greece. A majority of respondents complained about deficient legislation, lacking administrative structure, proper surveillance not taking place because of inept bureaucrats and widespread corruption. Public institutions would generally care little about the real situation in the Greek AR sector, but focus their activity on issuing unnecessary obligations which caused a lot of paperwork but contributed little to improving quality or implementing any kind of strategic plan for this promising niche of medical business. Clinical staff saw their sector as growing, because of the infrastructure built up so far and the continuous demand Greece is confronted with. There would be room for improvement, as several participants mentioned, especially by an effective quality control system which ensures that standards applied in the developed Western countries are implemented in Greece, too. In the long run, Greece would greatly profit from such a progress, because patients are likely to recommend institutions which are operating on a technically and ethically impeccable level.

Some of the respondent staff members cited managerial deficits, lack of funding, and deficient communication both inside the facility and between facilities and public authorities. The black list regarding medical authorities and administration in general contained incompetent and arbitrarily acting bureaucrats. Facilities were confronted with lengthy procedures and purposeless legal requirements and an outdated legislation. The state was failing to develop a coherent policy approach to promote the promising sector of assisted reproduction. In this respect, it is notable that professionals more frequently and intensely referred to shortcomings of the transport infrastructure than patients did. It is to be assumed that a greater percentage of patients don’t expect Greece to offer transport standards common in their home countries. Furthermore, patients’ situation is different in this regard: Roughly 93% of them travel to Greece’s for AR clusters, which are Athens, Piraeus, Crete (particularly Heraklion) and Thessaloniki. It’s more comfortable to reach these clusters from abroad than to travel between them inside Greece, Athens and Piraeus left aside.

Given these shortcomings, staff members showed an attitude which can be summarized with “against all odds, we’re performing well”. These odds include obstacles poor management cited above. Physicians argued that despite substantial difficulties they were ensuring high quality of treatments and a comforting environment inside their facilities, allowing them to be competitive on the international scale.

### Survey respondent characteristics

Among the patients who responded to the survey, 37 were from Italy, 17 each from Germany and France, 10 from UK, 9 each from Ireland and Netherlands, 4 from the USA, and the rest from various other European countries. In terms of gender, 64.1% of the respondents were females and 35.9% were males. In terms of marital status, 45.3% were currently married, 18.8% were cohabiting with someone, 14.8% were single, and 7% were in a same sex marriage/relationship. Finally, 18 respondents did not provide any answer regarding their relationship status. The largest group in terms of age was those belonging to 41–45 years (29.7%), followed by those who were 46–50 years old (18.8%). In the third place were those in 31–35 and 39–40 year groups (14.8% for each group). A few of the respondents were 26–30 years (3.9%), below 20 years (0.8%), or above 50 years (3.1%) old.

The majority of the participants either were currently in treatment (31.3%) or they had recently treatment within the last year (31.3%). In terms of AR facility location chosen, 37.5% of the patients went to Thessaloniki, 36.7% to Athens, and 11.7% to Crete. The remaining 18 respondents did not provide any answer. Based on self-report, the major types of treatment that the respondents sought in Greece were: Egg donation (38.2%), Embryo donation (20.9%), Tandem (10%), PGS aneuploidy screening (7.3%), IVF (5.5%), PGD (5.5%), IVF with donor sperm (4.5%), IUI with donor sperm (3.6%), and miscellaneous other treatments (4.5%).

### Satisfiers in AR tourism: analysis of patient responses

The patients were asked to declare the satisfaction derived from specific factors related to the assisted reproduction treatment they received. Their answers indicate that they were mostly satisfied with the climate (M = 3.80, SD = 0.809), respect of dignity of patient (M = 3.80, SD = 0.937), patient centeredness (M = 3.80, SD = 0.937), people friendliness (M = 3.79, SD = 0.781), and the waiting times (M = 3.77, SD = 0.774). On the contrary, they were least satisfied with the transportation (M = 2.77, SD = 1.077), outdoor activities (M = 2.78, SD = 1.059), accommodation (M = 2.85, SD = 0.795), accessibility to flights (M = 2.95, SD = 0.917) and the transparency provided (explanations, consent forms, documentation) (M = 2.96 SD = 0.995). It is clear that expectations related to satisfaction in RT are different from those in more traditional healthcare contexts. In the latter, clinical quality thumps up over relational aspects and non-clinical support services [39, 42, 44].

In order to understand the factors that stimulate someone to travel to Greece to receive treatment of assisted reproduction, the relevant item statements in the questionnaire related to patient satisfaction about Greece were subjected to factor analysis. Kaiser-Meyer-Olkin Measure of Sampling Adequacy was 0.675 (Approx. Chi-Square = 950.984). Bartlett’s Test of Sphericity yielded significant result. Principal Component Analysis was used for extraction. Varimax rotation was applied with Kaiser Normalization and the rotation converged in 9 iterations. The rotated component matrix is given below in Table 3:

**Table 3:** Factors determining satisfaction in reproductive tourism

	Component						
	1	2	3	4	5	6	7
Cost of travelling	-.058	.160	**.859**	.054	.219	.098	.213
Cost of staying (accommodation)	.031	.038	**.928**	.136	.030	.038	.088
Accessibility to the country	-.016	.127	.064	.220	.141	.062	**.822**
Cost of treatment	.071	-.339	.260	.061	.039	-.024	.739
Climate	**.738**	-.152	.083	.325	.049	-.148	-.128
Greece is a pleasant destination	**.829**	.072	-.146	.232	-.213	.004	-.054
Combining treatment with vacation	**.844**	-.120	-.065	-.170	-.088	-.081	.035
Image of Greece as medical tourism destination	**.729**	.099	.188	-.254	.029	.027	.202
Decision and desire of husband/wife	.006	.673	.045	-.160	.179	.090	-.047
Recommendation from relatives/friends/doctor	-.214	.612	-.009	.224	.024	.070	-.152
Mentality is close to mine	.204	**.741**	.044	.345	.102	.133	.197
Reasons related to religion	-.042	**.759**	.100	-.073	-.191	.241	-.016
The absence of similar clinics in my country	.006	.298	.016	**.754**	.145	.034	.366
Liberal legislative framework in Greece	.017	-.042	.325	**.722**	.204	-.019	.141
Quality of treatment	-.258	.258	-.001	.095	.034	**.761**	.035
Waiting times	.257	-.370	.439	.293	.011	.477	-.128
Reputation of doctor and/or clinic	.018	.212	.095	-.119	.075	**.818**	.031
Clinic accreditation	-.056	.232	.088	-.466	.547	.371	.246
Communication with clinic or clinic representatives in my language	-.116	.041	.179	.381	**.727**	.191	.064
Transparency granted during treatment	-.076	-.001	.072	.087	**.895**	-.071	.077

Note: All boldface entries are significant at p<0.05

The first seven factors explained a cumulative variance of 74.14%. Based on the above results, the factors that positively influence one to travel to Greece to receive assisted reproduction treatment may be approximately named as: Cost, accessibility, religious-spiritual reasons, liberal legislation, communication, quality and reputation of physicians/clinic, and combination of treatment with holiday.

### The case for and against Greece

Based on the descriptive analysis of the available data, the prevalent reasons for having treatment abroad have been identified as legislative restrictions in patients’ home country including treatment ineligibility for certain patient groups, costs, and perceived quality. The related data confirms that in countries with restrictive legislation (such as Germany, Italy [at least until 2014], Austria, Switzerland and Ireland), travelling to Greece for treatment has been a key reason. Patients from countries such as the UK and France, which allow treatment but pose strict eligibility criteria and have long waiting lines, have also rated “legislative restrictions in own country” and “absence of similar clinics in own country” as most important.

Treatment costs have been an encouraging factor mostly for patients from the USA, Romania, the Netherlands and Germany. Interestingly, Germans also rated costs as a discouraging factor for having treatment in Greece. Germans do have a more cost-friendly option for treatment (namely the Czech Republic), which is also geographically closer to Germany than Greece. Other countries that rated costs as a discouraging factor were, inter alia, the UK, Austria, France, Switzerland and the USA. Since treatment costs in these countries are actually higher, most probably patients compared –consciously or unconsciously – the costs to alternative destinations with lower costs, such as the Czech Republic or the Ukraine.

**Fig. 2:**
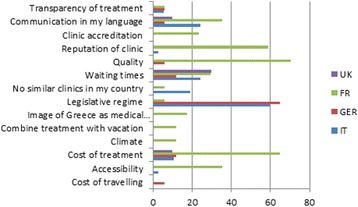
Encouraging factors to choose Greece as a destination for treatment (comparison of UK, FR, GER and IT patients). Factors favoring the choice of Greece as an AR destination

Perceived better quality has been an encouraging factor for choosing Greece as destination for treatment for patients from the UK, Ireland, Austria, Switzerland, New Zealand and France, although it was not rated highest by the most common group in our sample (namely Italians). As previously illustrated, most patients in this research were Italians, followed by Germans, French and UK patients. We attempted to adumbrate these segments more closely and compared their preferences. To be more precise, the factors that encouraged these patients most to choose Greece as a destination for assisted reproduction treatment vary, as Fig. 2 below depicts. Italians were convinced to choose Greece mostly for legislative reasons - a factor that has already been analyzed, and given legislative changes that took place in Italy in 2014 may be under transformation, followed by waiting times and communication in their language. For UK patients, waiting times play a decisive role. This is understandable, given the fact that in the UK patients might have waiting times of up to two years. German patients also seek treatment in Greece due to the liberal legislation. Here too it is well known that the restrictive German legislative regime forces Germans to seek treatment abroad.

**Fig. 3:**
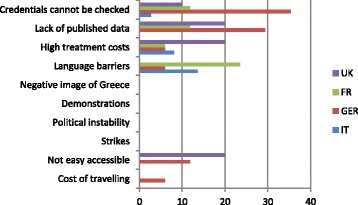
Discouraging factors to choose Greece as a destination for treatment (comparison of UK, FR, GER and IT patients). Factors disfavoring the choice of Greece as an AR destination

Looking at the same groups of patients, discouraging factors for choosing Greece as a destination for treatment of AR, are mainly lacking credentials and published data, language barriers and accessibility, as the Fig. 3 illustrates.

For Germans, lack of published data and credentials that cannot be checked are the most discouraging reasons for not choosing Greece as a destination. Given the fact that in countries such as the Czech Republic clinics must publish their data on their websites, including number of treatment cycles and success rates, Germans may be unwilling to trust a clinic without validated credentials. Accessibility seems to be another discouraging factor. Although there are daily flights from many German airports to the main Greek cities, travel costs are often high. The alternative for Germans to travel by car to a neighboring country as the Czech Republic is obviously easier and less costly.

The same is true for UK patients, who are also concerned about limited accessibility to Greece. In fact, low-cost airlines offer only a few connections to Greece. Patients from the UK apparently miss published data on the performance of clinics as well. Considering that the HFEA (Human Fertilization and Embryology Authority) provides exhaustive data for UK clinics, it is understandable that the lack of any similar information for Greece generates doubts. British patients guess that costs in Greece are high, although in reality they are much lower than in their own country. This feeling may result from the fact that they are comparing treatment costs with those in other countries, e.g., the Czech Republic, where they are indeed less.

French and Italian patients are most concerned with language barriers; Clinics with staff speaking their languages have consequently good chances for attracting these segments. This view is also supported by the fact that for Italians and French patients, the highest rated quality criteria are communication with the patient and patient centeredness (32.4 and 47.1% respectively). It seems like these groups value personalized treatment a lot, as well as transparency of treatment (24.3 and 41.2%, respectively) and cost transparency (18.9 and 41.2%, respectively). Italians care less about efficiency and after treatment care (each 2.7%) and French about safety and hygiene, while to both waiting times appear to be an important quality factor (13.5 and 29.4%) and for French, also scientific work of clinic seems to be an appealing quality factor (35.3%).

Waiting times, patient centeredness, efficiency and communication with the patient are ranked by UK patients as the most important quality criteria. As opposed to the Germans, UK patients do not rank latest technologies as a key criterion in defining quality; To UK patients, the latest technologies and communication with the patient are the second highest rated criteria in defining quality in their treatment (after transparency).

The results above gain more weight when looking at the ratings these groups gave their experiences after receiving treatment in Greece:

As previously seen, Italians identified the Italian legislative regime as the most encouraging reason for visiting Greece and language barriers as the most discouraging factor. Communication was rated as the highest quality criterion; only 2.7% were not satisfied with communication before treatment (vs. 32.4% who were satisfied) and 5.4% were not satisfied with the communication with the clinic staff (vs. 18.9% satisfied). The results show a satisfactory tendency, although a higher percentage of satisfaction could be an objective for clinics.

German patients, who as previously seem to care a lot about transparency and waiting times, seem to be satisfied enough with the latter (29.4%), but found transparency dismal (47.1% rated transparency provided as horrible, vs. 5.9% who rated it positively). Germans rated accessibility, staff friendliness and waiting times as being satisfactory overall. However, 11.8% found that the expertise of staff was not good (while 23.5% rated it the opposite).

For French patients, the most encouraging factor for seeking treatment in Greece was identified as the reputation and the quality of treatment abroad and in their understanding of quality, transparency had the highest rank. However, their evaluation of transparency was poor (only 5.9% expressed satisfaction, while 41.2% did not find it satisfactory). French patients, who also care greatly about communication in their language, were fairly satisfied with the communication with the clinic staff (29.4%), although the communication before treatment was rated as worse (29.4% rated it as horrible and only 17.6% as good). In addition the expertise and clinic infrastructure, both of which in a way reflect the scientific level of a clinic and matter to French audience, were rated by patients who had been to Greece as worse than expected. The clinic infrastructure was considered as good by only 17.6%, vs 23.5% who judged it “horrible”. Doctors’ expertise was rated as “good” by 17.6% and as “unsatisfactory” by 29.4% On the other hand, staff friendliness was rated the highest (35.3%). Considering that lack of transparency has also been a key finding of the qualitative research, it seems essential that Greece find solutions for enhancing transparency.

UK patients were encouraged to seek treatment in Greece mostly due to shorter waiting times but were discouraged by costs and deficient accessibility. So, these mixed responses may be an additional argument to come to grips with problems in the Greek transport system. The reason why Greece is yet attractive for patients from the UK may be that they could usually communicate directly with the physicians, who have learned English as their principal foreign language. As we can see from the results above, placing factors such as climate or combining treatment with vacation at the top of marketing activities aimed at foreign patients would be doomed to failure. Political instability, strikes, and demonstrations were not rated as of significant consequences. Among the groups compared above, German and UK patients rate strikes, demonstrations and political instability as important factors that discourage them from choosing Greece as a destination for treatment. But only in a slim majority of cases this results in choosing another destination as Greece for undergoing AR treatment.

**Fig. 4:**
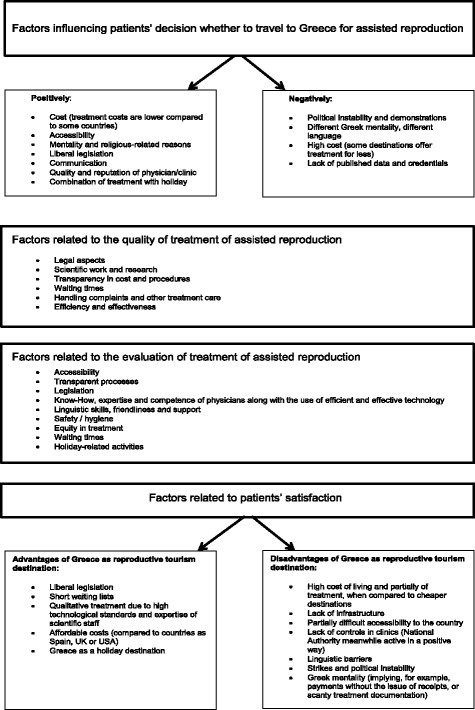
Summary of research findings

The summary of findings from the field research is presented in Fig. 4, diagrammatically.

Also, the summarized results of a Strengths-Weaknesses-Opportunities-Threats (SWOT) analysis, based on the findings of the study and in the light of the review of literature conducted by the authors, is presented in Table 4.

**Table 4:** SWOT analysis of reproductive tourism in Greece

STRENGTHS	OPPORTUNITIES
Liberal legislation Less ethical conflicts Short waiting lists Reputable physicians Globally comparable success rates Competitive prices compared to countries such as Spain, UK or USA Greece healthcare system is open to new technologies, with less barriers High investments in private clinics and excellent technological standards Comfortable location and good climate	Increasing social acceptance of ART Increased infertility, globally More complex legal status elsewhere (“loose bricks” for Greece) Lowering travel costs, making it possible to some traditionally inaccessible Grecian destinations New segments of reproductive tourists seeking a holistic experience
WEAKNESSES	THREATS
Economic crisis increases costs via increased taxation Political instability Lack of cost transparency Lacking transparency on results Lacking environmental policies Legal framework is not fully applied yet (although efforts of the National Authority flourish rapidly) Lacking organization on national level Staff training is not a standard requirement Expensive domestic connectivity within Greece Destination management programs are still developing Unstable linkages between healthcare and tourism industries.	Unstable economic situation in Greece leads to ongoing insecurity Political instability might affect legislation around ART Legal situation might change for better elsewhere and take away potential customers New entries are easy, this might lead to giants entering the market or increased (uncontrolled) rivalry Power of suppliers (pharma, donors, etc.) is very high Accessibility to Greece not granted for all High rivalry leads to lacking networking among the clinics

## Discussion

Greece’s infrastructure of assisted reproduction has to be seen in the broader context of the “European legislative mosaic” [1, 17]. The “Roman” group of countries in the EU, including Greece, has generous regulations as to legal conditions. The decision to choose a foreign destination in order to receive AR treatment is influenced by the legal constraints at home and the degree of liberalization in the country of choice [19]. As client groups, assisted reproductive tourists are not a monolithic whole. Lunt et al. identify three groups: medical refugees (a term the authors have adopted from Milstein & Smith, [34]; biotech pilgrims (adopted from Song, 2010); and those seeking cost advantages [28]. Evidently, the same strategy doesn’t attract these distinct segments.

The AR facilities around the world are competing on price, quality of service and accommodation, infrastructure and the overall attraction of their location [22]. People search overseas because, apart from possibly being outlawed, treatments in their home countries may be too costly [30]; or, the home country is lacking a private, soothing, and relaxing environment for assisted reproduction [8]. This research intended to clarify options for assisted tourism growth in the case of Greece. The findings could provide medical facilities with useful insights for further development of their services.

From an industry perspective, reproductive tourism can generate significant revenue for a country like Greece. Just over a thousand women went to Greece annually for assisted reproduction, which is far below the capacity of the system to handle demand. There are more than 60 facilities in Greece with special AR units, noted Pantos [32]. Further to the additional checks and balances placed by the Greek National Authority for Assisted Reproduction (GNAAR), reactivated in March 2014, the number of facilities have reduced to 44. The Authority is a national body that introduces and controls the scientific, legal, and moral framework in which all clinics and organizations related to assisted reproduction are functioning (http://eaiya.gov.gr/en/). As current evidence shows, capacities are far from exhausted. Yet, evidence from the interviews showed that patients’ expectations are only partly met. It is safe to state that Greece possesses a mix of advantages and disadvantages for reproductive tourism.

Greek clinics will profit from profiling and understanding their target segments. Identifying the opportunities in comparison with the competition, especially the legislative restrictions in other countries, is another first step. Understanding the patient-customer in depth, including their cultural backgrounds, is also essential and must occur before offers can be tailored to satisfy their needs and expectations.

Greece has growth potential as a destination for assisted reproduction treatments, but it lacks infrastructure and national strategy [31]. The introduction of partnerships with global medical organizations, including the promotion of medical tourism in target countries in the context of brand management, the establishment of quality assurance, licensing and control frameworks could become part of such a strategy. Furthermore, it would be desirable to leveraging networks to attract inbound volumes by enhancing alliances with medical and non-medical partners, such as medical tour operators [32]. Multilingual support, logistics and online consultations, as well as electronic patient record sharing are important as well.

Taking the discussion to the global context, assisted reproduction around the world has gained additional relevance through active governmental support [4, 10]. For example, the government of Korea set up a state-run research center, the Korean Medical Institute (KMI). Working together with, the Korean Tourism Organization and the Korean International Medical Association, it is expected to help expanding the reproductive assistance industry, including the attraction of foreign clients (Toyota, 2011, cit. [28]). In East Asia, Thailand has been a forerunner in these efforts. It managed to attract worldwide demand for assisted reproduction in the late 1990s [11]. In this country, hard hit by the economic crisis affecting the “tiger” economies, medical and touristic facilities re-directed their business strategies to assisted reproduction and medical tourism [28]. Japan would have the prerequisites to act as a hub for reproductive tourism as Thailand, but the domestic demand for medical services hinders this niche from developing further. So Japanese citizens are frequent customers of the facilities situated in the significantly smaller nations of Thailand and South Korea, with especially the latter enjoying high reputation for high-quality services.

Among Organization for Economic Co-operation and Development (OECD) member countries, the United States has the largest infrastructure in assisted reproduction [2, 23]. Kossoudij estimated that the U.S., including territories administered by Washington D.C., had at least 421 medical facilities qualifying for AR [25]. These findings are in line with data of the American Center for Disease Control for the year 1995, stating that of 60 million women of childbearing age, 13% had received infertility treatment [25]. According to Kossoduij, in 2001, there were 107,587 AR procedures performed in the U.S. These treatments led to 29,344 live births and 40,687 infants. According to Kossoudijs´ estimations, AR treatments have multiplied in the last two decades, with a 101% increase between 1996 and 2001 alone [25].

Germany and Austria, while being restrictive in some practices as PGD (preimplantation genetic diagnostics), have their insurances reimburse AR practices undertaken in other EU countries by people in other EU member countries, as long as they are legal in Austria and Germany [24]. So, these client groups constitute another interesting target group for Greek facilities (for those treatments allowed also in Austria and Germany), given that average treatment costs in Greece are typically less than in these countries. For Greece, there is a large and untapped potential for clients in cross-border medical care. Velissariou & Triantafyllos [43] calculated that about 900,000 Americans had travelled outside the US for medical care in 2013. According to the authors, 71% had saved more than € 2300 by travelling overseas and 12.7% even more than £ 10.000 [43].

Germany is among the EU member states with the most severe restrictions as to AR treatment, being among the few large member states not to permit PGD [17]. Generally, there is an uneven landscape inside the EU as to which kinds of AR treatments are allowed and which not. To get an overview of market perspectives it is useful to look at reimbursement regulations. This confirms the picture that the main target groups are to be sought in Germany, Austria, the Baltic countries and, to some extent Italy. There, citizens receive only partial compensation for IVF and Greece could reasonably benefit from these target segments.

## Conclusion

The present study may be seen as a first step in identifying why foreign patients choose Greece as their destination for undergoing assisted reproduction treatment, the advantages and disadvantages of Greece as an assisted reproduction tourism destination, and the strategies that can be applied to enhance Greece’s performance in this regard. It offered an examination of various issues surrounding assisted reproduction and reproductive tourism from three distinct discourse perspectives: legal-ethical discourse (e.g. the legal environment), demand discourse (e.g. the patients), and supply discourse (e.g. the clinical staff).

The study, in its empirical part, has been a field study. Insiders from the medical business were interviewed and assisted reproduction patients were surveyed. However, we are aware that this study is part of a larger picture that has not been drawn yet. Further studies with differing scope, methodology, participants, and locations may contribute to complete our idea of the emerging landscape of assisted reproductive tourism. Such a study could particularly focus on a decisive stage in the AR treatment cycle analyzed in this study. Since we limited the gathering of patient demographic data, cannot conclude whether the patients chosen are comparable in each group. Then, there are concerns about generalizing from small samples. We have avoided sweeping generalizations; despite this, some meaningful conclusions combining and contrasting the descriptive statistics with the extent literature were achieved. For instance, it is interesting to note that foreign patients tend to choose Greece for some of the most advanced of treatments such as egg donation and embryo donation. These are also two ‘controversial’ kinds of fertility treatments that either are forbidden, limited, or expensive in most other European countries, resulting in a unique basis of competitive advantage for Greece.

An overarching question in the literature is, to what extent patients’ expectations and the infrastructure plus quality of medical services are matching [3, 41]. As was shown in this study, this match is quite decisive in guiding a prospective patient’s choice of a particular facility in a particular country. So, it is worthwhile to investigate which factors are important for this match and to what extent. This study showed that Greece’s one key strength in RT is the success rates in treatment; its favorable AR legislation is a substantial opportunity, whereas the ongoing economic instability is constantly threatening the efforts of AR facilities to prove themselves as islands of good management in a crisis-stricken environment. As to costs, there’s a mixed picture because some cities and regions are less connected to the main traffic hubs than others.

The facilities themselves could achieve less demanding improvements more quickly: multilingual competence and transparency are some examples. In general, the staff need to be more cross-culturally competent. Language training means additional costs, but personnel may contribute by stepping up their own efforts. And there is no excuse for lacking transparency in dealing with their clients. Overcoming these shortcomings does not require additional funding, other than a change of mind and better communication.

A number of competitors, to mention only the Czech Republic as the most successful one in Europe, has done better and learned faster, despite starting from less favorable conditions 25 years ago. The Grecian AR system lacks organizational learning, despite the years put in the trade. Concerted approach by the State and the assisted reproduction sector could go much further. Greece should not continue to rely on its transient advantages. It turns out that others embrace Greece’s advantages, but avoid Greece’s shortcomings and so become more successful. Finally, assisted reproductive tourism is probably a way out to the current economic crisis [40]. Political forces and AR facilities in Greece should heed that call of the hour and make this sector live up to its full potential.

## Abbreviations

AR: Assisted reproduction
CBRC: Cross-border reproductive care
ESHRE: European society of human reproduction and embryology
GNAAR: Greek national authority for assisted reproduction
HFEA: Human fertilization and embryology authority
KMI: Korean medical institute
OECD: Organization for economic co-operation and development
RT: Reproductive tourism
